# Polymorphisms/Mutations in A-Kinase Anchoring Proteins (AKAPs): Role in the Cardiovascular System

**DOI:** 10.3390/jcdd5010007

**Published:** 2018-01-25

**Authors:** Santosh V. Suryavanshi, Shweta M. Jadhav, Bradley K. McConnell

**Affiliations:** Department of Pharmacological and Pharmaceutical Sciences, University of Houston College of Pharmacy, Texas Medical Center, Houston, TX 77204, USA; shwetamjadhav@gmail.com

**Keywords:** A-kinase anchoring proteins, single nucleotide polymorphisms, genetic mutations, cardiovascular diseases, protein kinase A

## Abstract

A-kinase anchoring proteins (AKAPs) belong to a family of scaffolding proteins that bind to protein kinase A (PKA) by definition and a variety of crucial proteins, including kinases, phosphatases, and phosphodiesterases. By scaffolding these proteins together, AKAPs build a “signalosome” at specific subcellular locations and compartmentalize PKA signaling. Thus, AKAPs are important for signal transduction after upstream activation of receptors ensuring accuracy and precision of intracellular PKA-dependent signaling pathways. Since their discovery in the 1980s, AKAPs have been studied extensively in the heart and have been proven essential in mediating cyclic adenosine monophosphate (cAMP)-PKA signaling. Although expression of AKAPs in the heart is very low, cardiac-specific knock-outs of several AKAPs have a noteworthy cardiac phenotype. Moreover, single nucleotide polymorphisms and genetic mutations in crucial cardiac proteins play a substantial role in the pathophysiology of cardiovascular diseases (CVDs). Despite the significant role of AKAPs in the cardiovascular system, a limited amount of research has focused on the role of genetic polymorphisms and/or mutations in AKAPs in increasing the risk of CVDs. This review attempts to overview the available literature on the polymorphisms/mutations in AKAPs and their effects on human health with a special focus on CVDs.

## 1. Introduction

The cardiovascular system, which is made up of heart, blood, and blood vessels, is essential for our survival [1]. The human heart provides oxygenated blood to itself and other tissues via a network of blood vessels supplying them with nutrients, and also removes carbon dioxide and other wastes from them. Proper blood circulation is required for effective regulation of these functions. Therefore, the continuous and flawless functioning of the heart becomes a very crucial indicator of normal cardiac physiology. Periodic beatings of the heart are regulated by a plethora of complex intracellular signaling cascades. Similarly, under acute (pregnancy, exercise, etc.) and chronic (pathophysiological stimulations) stress, the heart undergoes physiological and pathophysiological hypertrophy, respectively. Such changes in the anatomy of the heart at the cellular and molecular levels are governed by respective changes in the expression of hypertrophic transcription factors. Expression of essential transcription factors is also regulated by a complex network of intracellular signaling pathways [2,3].

Studying key proteins that are involved in regulating multiple intracellular signaling pathways has always interested cardiovascular scientists across the globe. By participating in the network of several signal transduction processes, these proteins play a central role in cardiac physiology and pathophysiology. A-kinase anchoring proteins (AKAPs) are one such type of proteins that belong to the family of scaffolding proteins. AKAPs have no intrinsic activity of their own, but their crucial function is to bind protein kinase A (PKA) and other signaling proteins [4,5]. PKA-mediated phosphorylation is critical for the physiological functioning of the heart [6]. AKAPs build a “signalosome” at various subcellular locations in the heart and regulate PKA-dependent signaling locally. Similarly, AKAPs speed up the PKA-mediated substrate phosphorylation by bringing all required components into close proximity with each other. Hence, by binding PKA and its substrates in the same scaffold, AKAPs monitor spatial and temporal PKA signaling [7].

AKAPs belong to a class of scaffolding proteins that are structurally not related to each other, but share key structural and/or signaling features. More than 70 AKAPs have been reported so far in humans. To be classified as an AKAP, proteins should have three properties:

(i) All AKAPs, though structurally different, have 14–18 α-helix amphipathic amino acid sequence that binds to regulatory subunits of PKA.
(ii) They have a targeting domain that tethers AKAPs to specific subcellular organelles, like mitochondria, the nucleus, and plasma membrane, among others.
(iii) Lastly, all AKAPs contain multiple binding domains by which they bind to other kinases than PKA, phosphatases, phosphodiesterases, and so on.

As the list of AKAPs is growing, the list of their binding partners is also rising. Hence, at a specific time, AKAPs bind only a subset of their binding partners, which depend on the cellular environment in which they are present [8]. Over the period of the last decade, several AKAPs have been identified in the cardiovascular system. So far, about 17 AKAPs have been discovered in the heart and the list will continue to grow further [9]. Efforts were made to understand the function of individual AKAPs by creating their specific knockouts. To mention a few, our laboratory had developed one such mouse model and has shown that gravin (AKAP12) mutant mice respond better to isoproterenol-induced stimulation than their wild-type counterparts with an improved cardiac profile [10]. Additionally, our unpublished data indicate that AKAP12 scaffolding is crucial for isoproterenol-mediated heart failure, and loss of function of AKAP12 scaffolding can act as a treatment strategy for heart failure [11]. Others have demonstrated that muscle-specific AKAPβ (mAKAPβ) scaffolds crucial signaling proteins around the nuclear envelope of cardiomyocytes that modulate cardiac remodeling. Conditional cardiac-specific deletion of mAKAPβ in adult mice was found to protect the heart from pressure overload and isoproterenol-induced cardiac stress [12]. The AKAP150 scaffold is also one of the promising targets for heart failure treatment. AKAP150 knock-out mice were prone to develop cardiomyopathy under pressure overload and expression of AKAP150 was significantly lower in failing mouse hearts [13]. 

Despite their importance in cardiac physiology and pathophysiology, there are very limited publications available showing the role of polymorphisms and/or mutations in AKAP in the cardiovascular system. In fact, our laboratory was the first to publish that single nucleotide polymorphisms in mAKAP alter binding propensities of phosphodiesterase4D3 (PDE4D3) and PKA to mAKAP [6]. Therefore, this review will attempt to address all the published literature on polymorphic and mutant AKAP, their role in the cardiovascular system, and their occurrence in other human diseases.

## 2. Role of AKAPs in Cardiovascular Physiology

AKAPs play a crucial role in human health and disease because of their control over local PKA signaling [3]. As PKA is a cAMP-dependent protein kinase, AKAPs are able to regulate the compartmentalization of cAMP and, therefore, the cAMP/PKA signaling pathway. The cAMP/PKA pathway is ubiquitous and a plethora of biological processes depend on it. Hence, AKAPs have an incomparable contribution in the physiology and pathophysiology of various human diseases [14,15]. In fact, pharmacological targeting of various AKAP-protein interactions has been proved beneficial in cardiovascular diseases, cancer, and other disorders, as shown in animal models of their respective diseases [5,14,16]. To be specific, AKAPs have become very promising drug targets for cardiovascular disease (CVD) due to their role in regulating and coordinating complex cardiac signaling pathways.

The literature on cardiac AKAPs reveals their importance in cardiac health and disease. AKAP-Lbc (AKAP13) is one such AKAP which is a pro-hypertrophic and pro-fibrotic AKAP, where hypertrophy is mediated via interleukin-6 (IL-6) and fibrosis is mediated via the Rho-guanine nucleotide exchange factor, respectively [17,18]. Deletion of AKAP13 in mice exhibited defective cardiac development and embryonic lethality [19]. AKAP150 (AKAP5, AKAP79) has multiple effects on heart function. On one hand, the loss of AKAP150 activates pathological cardiac hypertrophy by interfering with calcium dynamics and myocardial ionotropy, showing that its expression is critical for normal heart function [13]. On the other hand, by activating protein kinase C (PKC), AKAP150 mediates cardiac glucotoxicity [1]. Deletion of AKAP1 (AKAP121, AKAP149) leads to cardiac mitophagy and apoptosis after cardiac insult, thus indicating its role in the mitochondrial function in the heart [20]. mAKAP (AKAP6) is also a pro-hypertrophic AKAP that regulates pathological cardiac hypertrophic signaling pathways by directing the expression of transcription factors nuclear factor of activated T cells, cytoplasmic (NFATc), hypoxia-inducible factor 1 alpha subunit (HIF-1α), myocyte enhancer factor 2D (MEF2D), and histone deacetylase 4 (HDAC4). For this reason, AKAP6 is called the master scaffold for cardiac remodeling [21]. AKAP18 (AKAP15, AKAP7) acts as a nexus of signaling at the sarcoplasmic reticulum by regulating cardiac ionotropy. By binding to both protein phosphatase inhibitor-1 (I-1) and protein phosphatase-1 (PP-1), AKAP18 bi-directionally modulates phospholamban (PLB) phosphorylation and, thus, serves as a crucial regulator of cardiac function [22,23]. 

Yotiao (AKAP9) is an important AKAP regarding the electrophysiological coupling of the heart. By binding to the potassium channel (KCNQ), this AKAP maintains the slow outward potassium ion current. Specifically, mutations that prevent binding of yotiao and KCNQ result in long QT syndrome [24]. Studies in stem cell-derived cardiomyocytes indicate that AKAP10 (D-AKAP2) is essential in controlling the heart rate and rhythm [25]. Recently, it was shown that AKAP10 is crucial in erythropoietin signaling and heme biosynthesis at the outer mitochondrial membrane [26]. Cardiac phosphoinositide 3-kinase gamma (PI3Kγ) is an AKAP that binds phosphodiesterase3B (PDE3B) and phosphatidylinositol (3,4,5)-trisphosphate (PtdIns(3,4,5)P3) along with PKA regulating cAMP and PtdIns(3,4,5)P3 signaling. The crosstalk between cAMP and PtdIns(3,4,5)P3 is crucial in monitoring β-AR desensitization [27]. The catalytic subunit of PI3Kγ (p110γ) knockout mice exhibited significantly lower PDE3B activity leading to an abrupt increase in cAMP activity. Under stress, p110γ-null mice showed an uncontrolled rise in cAMP with the development of cardiomyopathy [28]. Gravin (AKAP12), on the other hand, is crucial in the β_2_-AR desensitization pathway. Our laboratory data revealed that gravin mutant mice performed better under acute isoproterenol stimulation as compared to their wild-type littermates [10]. Overall, these findings strongly suggest the role of AKAPs in cardiac physiology.

## 3. Polymorphisms/Mutations in AKAPs and CVDs

### 3.1. DAKAP2 (AKAP10)

Even though AKAPs have been implicated in cardiac disorders, there is a very limited number of publications on the impact of the genetics of AKAPs on CVDs. The first evidence of the direct interaction between polymorphic AKAP and heart disease came in 2003, where the I646V (A to G) polymorphism in D-AKAP2 (AKAP10) was found to be associated with changes in PR interval in the electrocardiography (ECG) of an older population [29]. The PR interval in ECG represents the time starting from the onset of atrial depolarization (the beginning of the P wave) until the onset of ventricular depolarization (the beginning of the QRS complex). It was reported in this study that homozygous valine variants of this old population showed a significantly lower PR intervals (slower atrial depolarization) than individuals who are homozygous for isoleucine. Experimental studies further suggested that this valine variant in AKAP10 had approximately three-fold higher binding to the PKA regulatory subunit RI alpha (PKA-RIα) than with isoleucine. AKAP10 is located in the outer mitochondrial membrane within the cardiomyocyte. Although PKA signaling with respect to AKAP10 is not known, AKAP10 contains a PDZ binding motif which could physically interact with transmembrane receptors and ion channels. Additionally, AKAP10 also contains two regulator of G protein signaling (RGS) domains by which AKAP10 might co-ordinate Gs activation and downstream PKA pathway [29]. Therefore, AKAP10 might scaffold a cardiac ion channel or exchanger. Thus, changes in the localization of PKA-RIα near this region, due to the polymorphic AKAP10, might have a significant impact on phosphorylation states of cardiac ion channel or exchanger leading to modulation of cardiac contraction. [29]. 

Lower PR intervals in homozygous valine population might be partially due to overactivation of ion channels or exchangers in the heart, and vice versa. Involvement of other possible unknown cell signaling pathways might be possible in the pathophysiology of functional AKAP10 variants. Due to the observed differences in PKA binding and respective changes in ECG due to AKAP10 polymorphism, research from this study showed, for the first time, that functional variants of AKAPs might have direct consequences on the etiology of CVDs. In subsequent studies, it was also shown that the I646V SNP of AKAP10 was common in 122 patients having coronary heart disease, as identified in the Heart and Soul Study (University of California, San Francisco, CA, USA; UCSF) [25]. It was revealed that homozygous carriers of the valine polymorphism had significantly higher heart rates than homozygous isoleucine carriers. Heart rate variability (HRV) and standard deviation of normal-to-normal (SDNN) R-R intervals, where R is the peak of a QRS complex, were found to be significantly lower in these patients. Low HRV and SDNN of R-R intervals are both indicators of sudden cardiac death in patients with some forms of CVDs. 

In another study, statistical data revealed that the effects of AKAP10 polymorphisms were independent of age, gender, race, and other heart-related risk parameters [25]. In the same study, and to understand the underlying mechanism, homozygous and heterozygous AKAP10 mutant mice were generated from AKAP10 mutant mouse embryonic stem cells (mESCs) having mutations in AKAP10’s PKA binding domain. Both in vivo and in vitro data from this study displayed increased contractile response to cholinergic agonists indicating that AKAP10 variants lead to the increase in vagal nerve sensitivity. Vagal inhibition of the heart was known to reduce pre-disposition to arrhythmia and sudden cardiac death. Hence, changes in vagal nerve sensitivity due to unknown molecular mechanisms contribute to the development of cardiac arrhythmia and death. 

Interestingly, it was also found that the 646I allele is exclusively common only in humans, while 15 other non-human animals, including chimpanzees, exhibit valine at 646 [25]. These results were further supported by a larger sample of a healthy middle-aged population of men and women having European ancestry. Association analysis on 1033 unrelated middle-aged men and women showed that participants with homozygous 646V had greater baseline HR and lower HRV values than homozygous 646I individuals. This analysis was done such that the results were not dependent on age, gender, smoking and drinking habits, exercise levels, and blood glucose [30]. In all the studies described above, the valine variant at position 646 was found to be 40% frequent, whereas the isoleucine variant was 60% frequent in all participants. Altogether, these results clearly suggest that the I646V functional variant of AKAP10 affects the sensitivity of heart’s pacemaker cells to sympathetic stimulation. In another study of a larger cohort of Japanese individuals, it was revealed that valine at 646 of AKAP10 was significantly associated with higher cases of myocardial infarction (MI) than in people with no history of hypercholesterolemia. The authors concluded that 646V was the risk factor for MI, although the molecular mechanism was not studied [31]. Thus, the AKAP10 I646V variant leads to abrupt heart rate (HR) and HRV changes along with increased risk of MI, possibly making healthy humans susceptible to an increased risk of arrhythmia and sudden cardiac death (Table 1).

In addition to the above-mentioned middle-aged and elderly population studies, the genetics of AKAP10 were also studied in newborns and infants. In polish newborns, 646V homozygous healthy infants showed longer QTc (corrected QT) intervals, but not out of the normal range, than isoleucine homozygous infants, suggesting a possible association between AKAP10 polymorphisms to the QTc interval [32]. Similarly, another study showed significantly higher mean blood pressure (BP) on the day one and the day three post-birth in Polish newborns carrying valine at the 646 position of AKAP10 as compared to isoleucine carriers [33]. Higher cholesterol cord blood concentration was also observed in Polish newborns at the time of birth due to I646V polymorphism in AKAP10. Homozygous valine carrier newborns (GG) had significantly increased levels of cholesterol than heterozygous (AG) and homozygous (AA) isoleucine carriers [34]. Another recent research report identified SNPs in AKAP10 in fetuses with ventricular septal defects and pulmonary stenosis, suggesting AKAP10 as a potential target for these cardiac conditions [35]. These reports suggest that functional AKAP10 variants affect cardiac parameters of newborns and infants, though the underlying molecular mechanisms were not studied. Taken together, AKAP10 polymorphisms play a significant role in increasing the susceptibility of humans of all age groups to develop specific CVDs (Table 1).

### 3.2. Yotiao (AKAP9)

Pharmacogenomics is a branch of pharmaceutical sciences which comprises studies to understand how genetics affect individual’s responses to drugs. Drug-induced prolongation of the QTc interval is a very serious adverse drug reaction causing drug withdrawal [36]. Nearly 1–5% of patients on anti-arrhythmic drug therapy have this severe complication. In one of the studies involving 1351 individuals it was noted that three novel AKAP polymorphisms were related to congenital arrhythmia. SNP in AKAP9 Gln3531Glu was found to be one the novel rare AKAP variants that was observed in congenital arrhythmia cases, which was also highly common in drug-induced Long-QT syndrome (LQTS) (Table 1) [37]. LQTS is one of the inheritable arrhythmia syndromes characterized by prolongation of the QT interval in ECG. LQTS patients undergo syncope, seizures, or cardiac arrest under physical or mental stress [38]. The QT interval indicates the time that is required for the heart muscle to send an electrical impulse through the ventricles and then recharge. LQTS generally occurs due to mutations in cardiac ion channels leading to a defective flow of ions in the heart. If the QT interval is longer than usual, then it will likely lead to a life-threatening ventricular arrhythmia called *torsade de pointes*.

The inheritable S1570L missense mutation in yotiao (AKAP9) was the first report of a disease-causing mutation in an AKAP [39]. Binding of slowly activating delayed rectifier potassium channel alpha subunit (KCNQ1) and yotiao is crucial for delayed rectifier current, which is important during the cardiac cycle of the human heart. Cardiac repolarization is mainly dependent upon rapid and slow delayed-rectifier potassium currents mediated by human ether-a-go-go-related (*hERG*) gene and the *KCNQ1* gene, respectively. KCNQ1 potassium channels become activated after the rapid current in late repolarization phase of the cardiac cycle and both these currents determine the length of action potential duration [40]. Phosphorylation of KCNQ1 is crucial for slow-activating delayed potassium current (I_Ks_). It was shown that PKA-mediated phosphorylation of KCNQ1 at serine 27 is critical for I_Ks_. Yotiao (AKAP9) effectively scaffolds PKA and protein phosphatase-1 (PP-1) to KCNQ1, maintaining its phosphorylation levels during upstream receptor activation [41]. The S1570L mutation was found in the KCNQ1-binding domain of yotiao, representing 2% of the clinically-robust LQTS-exhibiting patients. Molecular mechanistic studies showed that this mutation partially inhibits protein-protein interactions of KCNQ1-AKAP9, leading to decreased PKA-mediated phosphorylation of KCNQ1. Decreased phosphorylation of KCNQ1 subsequently resulted in prolonged repolarization of ventricular relaxation due to the elimination of the functional response of I_Ks_ to cAMP. This research displayed a direct link between genetic variants of AKAP and cardiac disease [39]. Furthermore, polymorphisms in AKAP9 were also found to be a modifier of LQTS in the South African population [42]. Four intronic AKAP9 polymorphisms, rs11772585 (C/T), rs7808587 (A/G), rs2282972 (C/T), and rs2961024 (A/C), were studied with or without the presence of the founder mutation, A341V. The rs11772585 T allele, along with the A431V founder mutation, increased the risk of cardiac events by more than two-fold, along with significantly increasing the severity of CVDs. The rs7808587 GG genotype polymorphism increased the risk of developing cardiac events by 74%. Interestingly, the rs2961024 GG genotype increased the QTc interval in the aging population in the absence of A341V mutation while the rs2282972 T allele altered the heart rate and QTc interval [42]. Thus, AKAP9, for the very first time, was shown to modify cardiac disease due to the presence of genetic polymorphisms (Table 1).

### 3.3. AKAP-Lbc (AKAP13)

Type 2 diabetes (T2D) is a risk factor for coronary artery disease as high blood glucose is known to increase the thickness of the arterial wall. AKAP-Lbc (AKAP13) is a cytoskeleton AKAP that binds to many proteins, including MEK1/2, extracellular signal-regulated kinase-1/2 (ERK1/2), PKCη, and protein kinase D (PKD). The mitogen-activated protein kinase (MAPK) signaling pathway is found to be common in both T2D and coronary artery disease [43]. As AKAP13 binds proteins that are involved in the MAPK pathway, SNPs in AKAP13 were found to be significantly linked with these diseases (Table 1) [43]. Other meta-analysis data on a Genome-Wide Association Study (GWAS) identified SNP in the AKAP13 gene from a Korean population for possible association with high blood pressure [44]. Researchers found that intronic rs11638762 (A/T) SNP in AKAP13, which lies in the GATA-3 binding site, was significantly reproduced in a duplication study done in a completely different group of individuals. AKAP13 scaffolds RhoA along with PKA to mediate activation of Rho family GTPase. Moreover, these GTPases are involved in cardiac hypertrophic signaling and the expression of AKAP13 is upregulated in hypertrophy. Changes in AKAP13 expression were also found to alter the expression of cardiac developmental genes, mainly myocyte enhancer factor 2C [44]. Neonatal death in AKAP10 knock-out mice due to thin-walled heart formation proved that AKAP13 expression is important for the development of the heart. Therefore, the authors hypothesized that the rs11638762 SNP might alter the expression of AKAP13 leading to defective cardiac development, which may have caused alterations in blood pressure levels [44].

### 3.4. Other AKAPs

Chronic kidney disease is one of the essential risk factors for CVDs. The intronic rs756009 A to G polymorphism in gravin (AKAP12) was associated with a higher risk of chronic kidney disease in Japanese patients with condition that also involves hypertension, diabetes, and high serum cholesterol [45]. However, the underlying molecular mechanisms of this AKAP12 polymorphism on the associated CVD were not studied (Table 1). The obesity-dependent parameters, body mass index (BMI) and waist-hip ratio (WHR), are proven risk factors for T2D and CVD. As such, a GWAS identified that the AKAP6 rs12885467 SNP was significantly associated with higher BMI [46]. Moreover, our unpublished data suggest that mAKAP polymorphisms might make humans more susceptible to cardiovascular diseases by altering cAMP/PKA signaling [47]. Additionally, AKAP7 Gln112Arg and AKAP6 Val839Ala SNPs were reported to be novel rare variants in congenital arrhythmia that were frequently found in drug-induced LQTS [37]. Overall, current evidence on polymorphisms/mutations in AKAPs strongly suggests a significant correlation of genetic variants of AKAPs with the pathophysiology of CVDs.

## 4. Polymorphisms/Mutations in AKAPs and Other Human Diseases

### 4.1. Neurological Disorders

AKAPs have been implicated in human diseases other than CVDs, especially diseases involving the brain, as well as other neurological disorders. Whole exome sequencing analysis of a Chinese family having idiopathic scoliosis (spinal deformity) identified the A2645C mutation in AKAP2 to be inherited in an autosomal dominant fashion [48]. It was also previously shown that disruption of AKAP2 expression, via de novo translocation, may contribute to Kallmann syndrome and bone anomalies [49]. Hence, AKAP2 may have a significant role in the pathogenesis of scoliosis. The K873R SNP in AKAP9 was linked to increased risk of developing schizophrenia in a Spanish population [50]. The AKAP9 SNPs rs144662445 (A/G, I to M) and rs149979685 (C/T, S to L) were found in seven African American Alzheimer’s disease patients and replicated successfully in the Alzheimer Disease Genetics Consortium (ADGC) population of 1037 cases and 1869 controls [51]. R3233C and R3832C variants of AKAP9 were identified and found to be prevalent in high-risk autism families [52]. In fact, AKAPs might have an incomparable role in etiology of autism spectrum disorders (ASDs). Single nucleotide polymorphisms in six AKAPs (AKAP7, AKAP10, AKAP11, microtubule-associated protein 2 (MAP2), moesin (MSN), and neurobeachin (NBEA)) were identified with possible association with ASDs. Especially, the SNP rs5918959 near the *MSN* gene displayed genome-wide significance [53]. The DNA copy variants in eight AKAP genes (AKAP5, AKAP8, AKAP9, AKAP10, AKAP13, MAP2, MSN, and NBEA) were also found in individuals with ASDs [53]. 

In yet other studies, AKAP5, shown to be present in all levels of the human brain, was observed to have a variable copy number in schizophrenia, bipolar disorder, and major depression behavior [54]. AKAP5 is involved in post-synaptic G-protein-coupled receptors (GPCRs)-mediated intracellular signaling which is known to have a role in modulating emotional behavior. Furthermore, studies involving AKAP5 polymorphisms revealed that the P100L polymorphism (rs2230491) affected the behavioral response of its carriers. Proline-carrying individuals had higher behavioral performance and working memory for emotional faces, while the less common leucine carriers exhibited greater control of their anger, but poor control of physical aggression [55,56]. Additional studies in the search of finding molecular mechanisms behind these behavioral changes showed that leucine carriers activated the anterior cingulate cortex (ACC), while proline homozygous individuals activated the orbitofrontal cortex (OFC) in the brain, as identified during emotional evaluation studies [56]. mAKAP (AKAP6) is one of the well-studied AKAPs in the heart. AKAP6 is expressed in cardiac muscle, skeletal muscle, and brain. In addition to its role in the heart, AKAP6 polymorphisms were found in GWAS studies to be related to various other human disorders. The intronic rs2383378 SNP was found to be associated with anorexia nervosa [57] and another intronic rs4296166 SNP was found to increase the risk of developing Alzheimer’s disease [58]. The rs17522122 SNP was found to be one of the genetic variants that significantly affect general fluid cognitive functioning in middle and old-age population of 53,949 individuals in the Cohorts for Heart and Aging Research in Genomic Epidemiology (CHARGE) consortium [59]. In a recent study, the rs17522122 T allele (found in UTR-3) SNP was associated with poor performance with respect to episodic memory in older populations, as identified in the Personality And Total Health (PATH) study [60]. Thus, polymorphisms in AKAPs were found to be significantly associated with a plethora of neurological disorders in humans.

### 4.2. Cancers

Genetic variants of AKAPs have been reported to be very common in the patient populations of various cancers. The whole genome and transcriptome sequencing data have shown that the AKAP2 gene is mutated in gastric and peritoneal metastatic cancer [61]. Yotiao (AKAP9) is a crucial AKAP with an important role in cell cycle progression, centrosome, and cell membrane function. Genetic mutations in AKAP9 are linked to a variety of cancers. Four AKAP9 mutations were associated with gastric cancer and 20 mutations were linked to colorectal cancer [62]. The single nucleotide polymorphisms M463I, 1389G > T and N2792S, 8375A > G in AKAP9 were found to increase the risk of familial breast cancer in German women [63]. The non-synonymous SNP M463I (rs6964587) in AKAP9 was also found to be very an important breast cancer susceptibility polymorphism [64]. The T allele of rs6964587 was frequent in African American, Asian, and European women. The AKAP9 M463I variant was also associated to increase the susceptibility of lung cancer in a large United Kingdom Caucasian population [65]. We previously discussed that the functional polymorphism I646V in D-AKAP2 (AKAP10) has a significant role in the cardiovascular system. In addition, heterozygous and homozygous expression of this valine variant in AKAP10 was also significantly increased in women with colorectal cancer as compared to isoleucine carriers [66]. Additionally, the valine AKAP10 variant carriers were also found to have increased risk of developing familial breast cancer [67]. Furthermore, the AKAP13 K526Q SNP, along with I646V in AKAP10, further augmented the risk for breast cancer [67]. Since AKAP13 plays a crucial scaffolding role in Rho GTPase intracellular signaling, SNPs in this protein may also affect various cancers. The polymorphisms R494W, K526Q, N1086D, and G2461S in AKAP13 were all found in familial breast cancer patients with K526Q having the highest association among all of these SNPs [68]. SNPs in gravin (AKAP12) were also identified to be significantly associated with breast cancer risk and osteosarcoma [69,70]. In conclusion, mutated and/or polymorphic AKAPs have been associated with the increased risk of different types of cancers in humans.

### 4.3. Other Human Disorders

**Figure 1:**
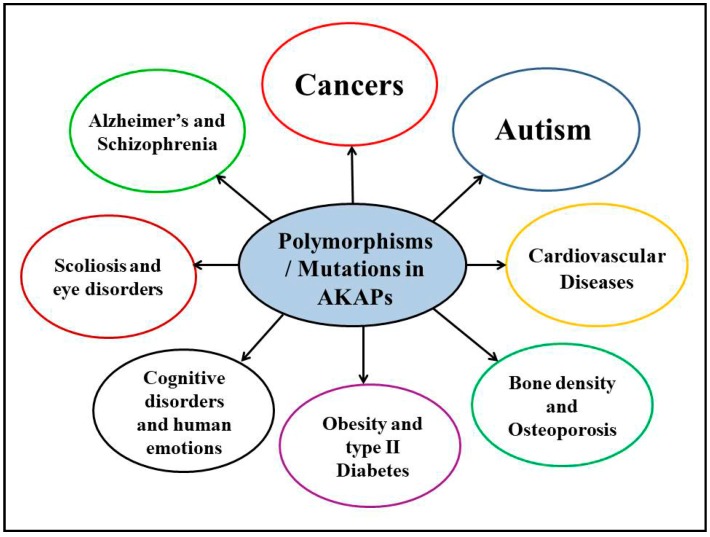
Genetics of AKAPs and human diseases.

In addition to neurological diseases and cancers, polymorphic AKAPs were also associated with a few other human disorders. The heterozygous mutation in AKAP4 (887G > A; G296N) was found in men with low sperm motility, a condition known as asthenozoospermia [71]. AKAP4 is a testis-specific AKAP and its removal in mice leads to reduced sperm motility, resulting in infertility. As this mutation was absent in matched control males, this AKAP4 mutation might be the probable cause of male infertility [71]. Another report found that mutations in AKAP4 may cause sperm fibrous sheath dysplasia [72]. In another study, SNPs in the AKAP ezrin were found to increase the risk and development of age-related cataracts [73]. Finally, the AKAP11 genetic locus was identified in the GWAS to be significantly associated with osteoporosis [74]. In summary, genetic variations in AKAPs have also been significantly implicated in other human diseases along with CVDs (Figure 1).

## 5. Conclusions 

AKAPs are a group of proteins that scaffold PKA by definition. Along with binding and localizing PKA to specific subcellular compartments, AKAPs also scaffold other proteins in the vicinity of PKA, thus efficiently regulating intracellular PKA-dependent signaling cascades. Additional proteins that AKAPs bind include a variety of other kinases, phosphatases, phosphodiesterases, GPCRs, and signaling proteins. Moreover, AKAPs have been reported to play a pivotal role in both the physiology and the pathophysiology of human diseases. However, the role of genetics in AKAPs with respect to human diseases has been underappreciated, especially in the cardiovascular system where polymorphisms/mutations in AKAPs have been shown to increase the risk of developing various CVDs. Molecular mechanisms behind increasing the risk of CVDs were also studied in certain candidate AKAPs. Similarly, numerous SNPs were identified that increased the susceptibility of individuals to develop cancers, brain disorders, male infertility, and eye disorders. We believe that the majority of the literature only mentions the SNPs and/or mutations in AKAPs with their respective human disorders. However, we think that genetic variants in AKAPs should also be further studied with respect to the molecular mechanisms involved in these disorders. Although very few attempts were made in studying the mechanistic pathways in increasing the risk of developing cardiovascular disease, a substantial amount of additional research should be done with respect to CVDs, as well as other human diseases.

## Figures and Tables

**Table 1:** Polymorphisms/mutations in AKAPs and CVDs.

AKAPs	SNPs/Mutations and Heart Disease	Reference
AKAP6	SNP Val839Ala; cardiac arrhythmia	[37]
	SNP rs12885467; higher BMI	[46]
AKAP7	SNP Gln112Arg; cardiac arrhythmia	[37]
AKAP9	SNP Gln3531Glu; cardiac arrhythmia	[37]
	Mutation Ser1570Leu; long-QT syndrome	[39]
	Four SNPs; long-QT Syndrome Type 1	[42]
AKAP10	SNP Ile646Val; decrease in PR interval	[29]
	Mutations; cardiac arrhythmia	[25]
	SNP Ile646Val; myocardial infarction	[31]
	SNP Ile646Val; blood pressure	[33]
	SNP Ile646Val; hypercholesterolemia	[34]
	SNP Ile646Val; heart rate variability	[30]
	SNP Ile646Val; long QTc interval length	[32]
	Copy number variations; ventricular septal defects	[35]
AKAP12	SNP with multiple alleles; chronic kidney disease	[45]
AKAP13	Genetic locus; coronary artery disease	[43]
	SNP; high blood pressure	[44]

## References

[1] Diviani D., Reggi E., Arambasic M., Caso S., Maric D. (2016). Emerging roles of A-kinase anchoring proteins in cardiovascular pathophysiology. Biochim. Biophys. Acta.

[2] Shimizu I., Minamino T. (2016). Physiological and pathological cardiac hypertrophy. J. Mol. Cell Cardiol..

[3] Rababa’h A., Singh S., Suryavanshi S.V., Altarabsheh S.E., Deo S.V., McConnell B.K. (2014). Compartmentalization role of A-kinase anchoring proteins (AKAPs) in mediating protein kinase A (PKA) signaling and cardiomyocyte hypertrophy. Int. J. Mol. Sci..

[4] Li Z., Singh S., Suryavanshi S.V., Ding W., Shen X., Wijaya C.S., Gao W.D., McConnell B.K. (2017). Force development and intracellular Ca^2+^ in intact cardiac muscles from gravin mutant mice. Eur. J. Pharmacol..

[5] Carnegie G.K., Means C.K., Scott J.D. (2009). A-kinase anchoring proteins: From protein complexes to physiology and disease. IUBMB Life.

[6] Rababa’h A., Craft J.W., Wijaya C.S., Atrooz F., Fan Q., Singh S., Guillory A.N., Katsonis P., Lichtarge O., McConnell B.K. (2013). Protein kinase A and phosphodiesterase-4D3 binding to coding polymorphisms of cardiac muscle anchoring protein (mAKAP). J. Mol. Biol..

[7] Kapiloff M.S., Chandrasekhar K.D. (2011). A-kinase anchoring proteins: Temporal and spatial regulation of intracellular signal transduction in the cardiovascular system. J. Cardiovasc. Pharmacol..

[8] Wong W., Scott J.D. (2004). AKAP signalling complexes: Focal points in space and time. Nat. Rev. Mol. Cell Biol..

[9] Kritzer M.D., Li J., Dodge-Kafka K., Kapiloff M.S. (2012). AKAPs: The architectural underpinnings of local camp signaling. J. Mol. Cell Cardiol..

[10] Guillory A.N., Yin X., Wijaya C.S., Diaz Diaz A.C., Rababa’h A., Singh S., Atrooz F., Sadayappan S., McConnell B.K. (2013). Enhanced cardiac function in gravin mutant mice involves alterations in the beta-adrenergic receptor signaling cascade. PLoS ONE.

[11] McConnell B., Suryavanshi S., Fa’ak F., Diaz A.D., Singh S. (2016). Disruption of gravin’s scaffolding protects against isoproterenol induced heart failure. FASEB J..

[12] Kritzer M.D., Li J., Passariello C.L., Gayanilo M., Thakur H., Dayan J., Dodge-Kafka K., Kapiloff M.S. (2014). The scaffold protein muscle A-kinase anchoring protein beta orchestrates cardiac myocyte hypertrophic signaling required for the development of heart failure. Circ. Heart Fail..

[13] Li L., Li J., Drum B.M., Chen Y., Yin H., Guo X., Luckey S.W., Gilbert M.L., McKnight G.S., Scott J.D. (2017). Loss of AKAP150 promotes pathological remodelling and heart failure propensity by disrupting calcium cycling and contractile reserve. Cardiovasc. Res..

[14] Dema A., Perets E., Schulz M.S., Deak V.A., Klussmann E. (2015). Pharmacological targeting of AKAP-directed compartmentalized camp signalling. Cell Signal..

[15] Michel J.J., Scott J.D. (2002). AKAP mediated signal transduction. Annu. Rev. Pharmacol. Toxicol..

[16] Troger J., Moutty M.C., Skroblin P., Klussmann E. (2012). A-kinase anchoring proteins as potential drug targets. Br. J. Pharmacol..

[17] Cavin S., Maric D., Diviani D. (2014). A-kinase anchoring protein-Lbc promotes pro-fibrotic signaling in cardiac fibroblasts. Biochim. Biophys. Acta.

[18] Del Vescovo C.D., Cotecchia S., Diviani D. (2013). A-kinase-anchoring protein-Lbc anchors ikappab kinase beta to support interleukin-6-mediated cardiomyocyte hypertrophy. Mol. Cell Biol..

[19] Mayers C.M., Wadell J., McLean K., Venere M., Malik M., Shibata T., Driggers P.H., Kino T., Guo X.C., Koide H. (2010). The rho guanine nucleotide exchange factor AKAP13 (brx) is essential for cardiac development in mice. J. Biol. Chem..

[20] Schiattarella G.G., Cattaneo F., Pironti G., Magliulo F., Carotenuto G., Pirozzi M., Polishchuk R., Borzacchiello D., Paolillo R., Oliveti M. (2016). AKAP1 deficiency promotes mitochondrial aberrations and exacerbates cardiac injury following permanent coronary ligation via enhanced mitophagy and apoptosis. PLoS ONE.

[21] Passariello C.L., Li J., Dodge-Kafka K., Kapiloff M.S. (2015). mAKAP—A master scaffold for cardiac remodeling. J. Cardiovasc. Pharmacol..

[22] Redden J.M., Dodge-Kafka K.L. (2011). AKAP phosphatase complexes in the heart. J. Cardiovasc. Pharmacol..

[23] Singh A., Redden J.M., Kapiloff M.S., Dodge-Kafka K.L. (2011). The large isoforms of A-kinase anchoring protein 18 mediate the phosphorylation of inhibitor-1 by protein kinase A and the inhibition of protein phosphatase 1 activity. Mol. Pharmacol..

[24] Li Y., Chen L., Kass R.S., Dessauer C.W. (2012). The A-kinase anchoring protein Yotiao facilitates complex formation between adenylyl cyclase type 9 and the I_Ks_ potassium channel in heart. J. Biol. Chem..

[25] Tingley W.G., Pawlikowska L., Zaroff J.G., Kim T., Nguyen T., Young S.G., Vranizan K., Kwok P.Y., Whooley M.A., Conklin B.R. (2007). Gene-trapped mouse embryonic stem cell-derived cardiac myocytes and human genetics implicate AKAP10 in heart rhythm regulation. Proc. Natl. Acad. Sci. USA.

[26] Chung J., Wittig J.G., Ghamari A., Maeda M., Dailey T.A., Bergonia H., Kafina M.D., Coughlin E.E., Minogue C.E., Hebert A.S. (2017). Erythropoietin signaling regulates heme biosynthesis. Elife.

[27] Perrino C., Schroder J.N., Lima B., Villamizar N., Nienaber J.J., Milano C.A., Naga Prasad S.V. (2007). Dynamic regulation of phosphoinositide 3-kinase-γ activity and β-adrenergic receptor trafficking in end-stage human heart failure. Circulation.

[28] Perino A., Ghigo A., Ferrero E., Morello F., Santulli G., Baillie G.S., Damilano F., Dunlop A.J., Pawson C., Walser R. (2011). Integrating cardiac PIP3 and cAMP signaling through a PKA anchoring function of p110γ. Mol. Cell.

[29] Kammerer S., Burns-Hamuro L.L., Ma Y., Hamon S.C., Canaves J.M., Shi M.M., Nelson M.R., Sing C.F., Cantor C.R., Taylor S.S. (2003). Amino acid variant in the kinase binding domain of dual-specific a kinase-anchoring protein 2: A disease susceptibility polymorphism. Proc. Natl. Acad. Sci. USA.

[30] Neumann S.A., Tingley W.G., Conklin B.R., Shrader C.J., Peet E., Muldoon M.F., Jennings J.R., Ferrell R.E., Manuck S.B. (2009). AKAP10 (i646v) functional polymorphism predicts heart rate and heart rate variability in apparently healthy, middle-aged European-Americans. Psychophysiology.

[31] Nishihama K., Yamada Y., Matsuo H., Segawa T., Watanabe S., Kato K., Yajima K., Hibino T., Yokoi K., Ichihara S. (2007). Association of gene polymorphisms with myocardial infarction in individuals with or without conventional coronary risk factors. Int. J. Mol. Med..

[32] Loniewska B., Kaczmarczyk M., Clark J.S., Goracy I., Horodnicka-Jozwa A., Ciechanowicz A. (2015). Association of functional genetic variants of A-kinase anchoring protein 10 with QT interval length in full-term polish newborns. Arch. Med. Sci..

[33] Loniewska B., Kaczmarczyk M., Clark J.S., Binczak-Kuleta A., Adler G., Kordek A., Horodnicka-Jozwa A., Dawid G., Rudnicki J., Ciechanowicz A. (2013). Association of 1936a > g in AKAP10 (A-kinase anchoring protein 10) and blood pressure in polish full-term newborns. Blood Press.

[34] Loniewska B., Kaczmarczyk M., Clark J.S., Kordek A., Ciechanowicz A. (2013). Polymorphism 1936a > g in the AKAP10 gene (encoding A-kinase-anchoring protein 10) is associated with higher cholesterol cord blood concentration in polish full-term newsborns. J. Perinat. Med..

[35] Fu F., Deng Q., Lei T.Y., Li R., Jing X.Y., Yang X., Liao C. (2017). Clinical application of SNP array analysis in fetuses with ventricular septal defects and normal karyotypes. Arch. Gynecol. Obstet..

[36] Wilke R.A., Lin D.W., Roden D.M., Watkins P.B., Flockhart D., Zineh I., Giacomini K.M., Krauss R.M. (2007). Identifying genetic risk factors for serious adverse drug reactions: Current progress and challenges. Nat. Rev. Drug Discov..

[37] Ramirez A.H., Shaffer C.M., Delaney J.T., Sexton D.P., Levy S.E., Rieder M.J., Nickerson D.A., George A.L., Roden D.M. (2013). Novel rare variants in congenital cardiac arrhythmia genes are frequent in drug-induced torsades de pointes. Pharmacogenomics J..

[38] Schwartz P.J., Crotti L., Insolia R. (2012). Long-QT syndrome: From genetics to management. Circ. Arrhythm. Electrophysiol..

[39] Chen L., Marquardt M.L., Tester D.J., Sampson K.J., Ackerman M.J., Kass R.S. (2007). Mutation of an A-kinase-anchoring protein causes long-QT syndrome. Proc. Natl. Acad. Sci. USA.

[40] Moss A.J., Kass R.S. (2005). Long QT syndrome: From channels to cardiac arrhythmias. J. Clin. Investig..

[41] Chen L., Sampson K.J., Kass R.S. (2016). Cardiac delayed rectifier potassium channels in health and disease. Card. Electrophysiol. Clin..

[42] De Villiers C.P., van der Merwe L., Crotti L., Goosen A., George A.L., Schwartz P.J., Brink P.A., Moolman-Smook J.C., Corfield V.A. (2014). AKAP9 is a genetic modifier of congenital long-QT syndrome type 1. Circ. Cardiovasc. Genet..

[43] Dong C., Tang L., Liu Z., Bu S., Liu Q., Wang Q., Mai Y., Wang D.W., Duan S. (2014). Landscape of the relationship between type 2 diabetes and coronary heart disease through an integrated gene network analysis. Gene.

[44] Hong K.W., Lim J.E., Oh B. (2011). A regulatory SNP in AKAP13 is associated with blood pressure in Koreans. J. Hum. Genet..

[45] Yoshida T., Kato K., Yokoi K., Oguri M., Watanabe S., Metoki N., Yoshida H., Satoh K., Aoyagi Y., Nozawa Y. (2009). Association of gene polymorphisms with chronic kidney disease in Japanese individuals. Int. J. Mol. Med..

[46] Horikoshi M., Mgi R., van de Bunt M., Surakka I., Sarin A.P., Mahajan A., Marullo L., Thorleifsson G., Hgg S., Hottenga J.J. (2015). Discovery and fine-mapping of glycaemic and obesity-related trait loci using high-density imputation. PLoS Genet..

[47] Suryavanshi S., Jadhav S., Anderson K., Katsonis P., Lichtarge O., McConnell B.K. (2017). Abstract 24010: Muscle-specific A-kinase anchoring protein polymorphisms pre-dispose humans to cardiovascular diseases by affecting cyclic AMP/PKA signaling. Circulation.

[48] Li W., Li Y., Zhang L., Guo H., Tian D., Li Y., Peng Y., Zheng Y., Dai Y., Xia K. (2016). AKAP2 identified as a novel gene mutated in a Chinese family with adolescent idiopathic scoliosis. J. Med. Genet..

[49] Panza E., Gimelli G., Passalacqua M., Cohen A., Gimelli S., Giglio S., Ghezzi C., Sparatore B., Heye B., Zuffardi O. (2007). The breakpoint identified in a balanced de novo translocation t(7;9)(p14.1;q31.3) disrupts the A-kinase (PRKA) anchor protein 2 gene (AKAP2) on chromosome 9 in a patient with kallmann syndrome and bone anomalies. Int. J. Mol. Med..

[50] Suarez-Rama J.J., Arrojo M., Sobrino B., Amigo J., Brenlla J., Agra S., Paz E., Brion M., Carracedo A., Paramo M. (2015). Resequencing and association analysis of coding regions at twenty candidate genes suggest a role for rare risk variation at AKAP9 and protective variation at nrxn1 in schizophrenia susceptibility. J. Psychiatr. Res..

[51] Logue M.W., Schu M., Vardarajan B.N., Farrell J., Bennett D.A., Buxbaum J.D., Byrd G.S., Ertekin-Taner N., Evans D., Foroud T. (2014). Two rare AKAP9 variants are associated with Alzheimer’s disease in African Americans. Alzheimer’s Dement..

[52] Matsunami N., Hensel C.H., Baird L., Stevens J., Otterud B., Leppert T., Varvil T., Hadley D., Glessner J.T., Pellegrino R. (2014). Identification of rare DNA sequence variants in high-risk autism families and their prevalence in a large case/control population. Mol. Autism.

[53] Poelmans G., Franke B., Pauls D.L., Glennon J.C., Buitelaar J.K. (2013). AKAPs integrate genetic findings for autism spectrum disorders. Transl. Psychiatry.

[54] Wilson G.M., Flibotte S., Chopra V., Melnyk B.L., Honer W.G., Holt R.A. (2006). DNA copy-number analysis in bipolar disorder and schizophrenia reveals aberrations in genes involved in glutamate signaling. Hum. Mol. Genet..

[55] Richter S., Gorny X., Machts J., Behnisch G., Wustenberg T., Herbort M.C., Munte T.F., Seidenbecher C.I., Schott B.H. (2013). Effects of AKAP5 pro100leu genotype on working memory for emotional stimuli. PLoS ONE.

[56] Richter S., Gorny X., Marco-Pallares J., Kramer U.M., Machts J., Barman A., Bernstein H.G., Schule R., Schols L., Rodriguez-Fornells A. (2011). A potential role for a genetic variation of AKAP5 in human aggression and anger control. Front. Hum. Neurosci..

[57] Wang K., Zhang H., Bloss C.S., Duvvuri V., Kaye W., Schork N.J., Berrettini W., Hakonarson H., Price Foundation Collaborative Group (2011). A genome-wide association study on common SNPs and rare CNVs in anorexia nervosa. Mol. Psychiatry.

[58] Seshadri S., Fitzpatrick A.L., Ikram M.A., DeStefano A.L., Gudnason V., Boada M., Bis J.C., Smith A.V., Carassquillo M.M., Lambert J.C. (2010). Genome-wide analysis of genetic loci associated with alzheimer disease. JAMA.

[59] Davies G., Armstrong N., Bis J.C., Bressler J., Chouraki V., Giddaluru S., Hofer E., Ibrahim-Verbaas C.A., Kirin M., Lahti J. (2015). Genetic contributions to variation in general cognitive function: A meta-analysis of genome-wide association studies in the charge consortium (*N* = 53,949). Mol. Psychiatry.

[60] Andrews S.J., Das D., Anstey K.J., Easteal S. (2017). Association of AKAP6 and mir2113 with cognitive performance in a population-based sample of older adults. Genes Brain Behav..

[61] Zhang J., Huang J.Y., Chen Y.N., Yuan F., Zhang H., Yan F.H., Wang M.J., Wang G., Su M., Lu G. (2015). Whole genome and transcriptome sequencing of matched primary and peritoneal metastatic gastric carcinoma. Sci. Rep..

[62] Jo Y.S., Kim M.S., Yoo N.J., Lee S.H. (2016). Frameshift mutations of AKAP9 gene in gastric and colorectal cancers with high microsatellite instability. Pathol. Oncol. Res..

[63] Frank B., Wiestler M., Kropp S., Hemminki K., Spurdle A.B., Sutter C., Wappenschmidt B., Chen X., Beesley J., Hopper J.L. (2008). Association of a common AKAP9 variant with breast cancer risk: A collaborative analysis. J. Natl. Cancer Inst..

[64] Milne R.L., Burwinkel B., Michailidou K., Arias-Perez J.I., Zamora M.P., Menendez-Rodriguez P., Hardisson D., Mendiola M., Gonzalez-Neira A., Pita G. (2014). Common non-synonymous SNPs associated with breast cancer susceptibility: Findings from the breast cancer association consortium. Hum. Mol. Genet..

[65] Rudd M.F., Webb E.L., Matakidou A., Sellick G.S., Williams R.D., Bridle H., Eisen T., Houlston R.S., Consortium G. (2006). Variants in the *GH*-*IGF* axis confer susceptibility to lung cancer. Genome Res..

[66] Wang M., Zhang D., Wang R., Rui Y., Zhou J., Wang R., Zhou B., Huang X., Yang L., Li Y. (2013). A-kinase anchoring proteins 10 expression in relation to 2073a/g polymorphism and tumor progression in patients with colorectal cancer. Pathol. Oncol. Res..

[67] Wirtenberger M., Schmutzhard J., Hemminki K., Meindl A., Sutter C., Schmutzler R.K., Wappenschmidt B., Kiechle M., Arnold N., Weber B.H. (2007). The functional genetic variant ile646val located in the kinase binding domain of the A-kinase anchoring protein 10 is associated with familial breast cancer. Carcinogenesis.

[68] Wirtenberger M., Tchatchou S., Hemminki K., Klaes R., Schmutzler R.K., Bermejo J.L., Chen B., Wappenschmidt B., Meindl A., Bartram C.R. (2006). Association of genetic variants in the rho guanine nucleotide exchange factor AKAP13 with familial breast cancer. Carcinogenesis.

[69] Kresse S.H., Rydbeck H., Skarn M., Namlos H.M., Barragan-Polania A.H., Cleton-Jansen A.M., Serra M., Liestol K., Hogendoorn P.C., Hovig E. (2012). Integrative analysis reveals relationships of genetic and epigenetic alterations in osteosarcoma. PLoS ONE.

[70] Sun Y., Ye C., Guo X., Wen W., Long J., Gao Y.T., Shu X.O., Zheng W., Cai Q. (2016). Evaluation of potential regulatory function of breast cancer risk locus at 6q25.1. Carcinogenesis.

[71] Visser L., Westerveld G.H., Xie F., van Daalen S.K., van der Veen F., Lombardi M.P., Repping S. (2011). A comprehensive gene mutation screen in men with asthenozoospermia. Fertil. Steril..

[72] Baccetti B., Collodel G., Estenoz M., Manca D., Moretti E., Piomboni P. (2005). Gene deletions in an infertile man with sperm fibrous sheath dysplasia. Hum. Reprod..

[73] Lin Q., Zhou N., Zhang N., Zhu B., Hu S., Zhou Z., Qi Y. (2013). Genetic variations and polymorphisms in the ezrin gene are associated with age-related cataract. Mol. Vis..

[74] Zhang L., Choi H.J., Estrada K., Leo P.J., Li J., Pei Y.F., Zhang Y., Lin Y., Shen H., Liu Y.Z. (2014). Multistage genome-wide association meta-analyses identified two new loci for bone mineral density. Hum. Mol. Genet..

